# Enjoyment of AI-generated stories blending art and science: impact on preschoolers’ pro-environmental attitudes

**DOI:** 10.3389/fpsyg.2025.1579510

**Published:** 2025-07-23

**Authors:** Jiaqi Chen

**Affiliations:** School of Preschool Education, Jiangmen Preschool Education College, Jiangmen, China

**Keywords:** AI-generated stories, preschoolers, environmental sensitivity, pro-environmental behavior, storytelling enjoyment, Chinese preschool education

## Abstract

**Background:**

This study explores the impact of artificial intelligence (AI)-generated stories that blend art and science on the development of pro-environmental attitudes and behaviors in preschoolers. AI-generated stories represent an innovative educational tool that leverages technology to create engaging, inclusive, and educational narratives. These stories can introduce young children to complex environmental concepts, fostering both cognitive understanding and emotional engagement with nature.

**Hypotheses:**

The research examines three key hypotheses: (1) enjoyment of AI-generated stories will positively influence preschoolers’ pro-environmental behavior, (2) enjoyment of AI-generated stories will positively influence preschoolers’ environmental sensitivity, and (3) environmental sensitivity will mediate the relationship between story enjoyment and pro-environmental behavior.

**Methods:**

The study included a total sample of 120 Chinese preschoolers from Upper Kindergarten (大班) in Jiangmen City. Initially, children were introduced to AI-generated stories. After listening to the stories, their level of enjoyment was assessed. At the second time point, environmental sensitivity was measured through a game. At the third and final time point, children’s pro-environmental behavior was assessed through a task designed to evaluate their actions and attitudes related to environmental conservation.

**Results:**

Findings from this study suggest that preschoolers who enjoy AI-generated stories are more likely to display pro-environmental attitudes and behaviors, such as recycling and conserving resources. Furthermore, environmental sensitivity—a personal trait characterized by an emotional and cognitive connection to nature—was found to be a significant mediator in this relationship. Children who exhibit higher levels of environmental sensitivity are more likely to internalize the environmental messages embedded in the stories, translating enjoyment into meaningful, real-world actions.

**Discussion:**

By combining the creative elements of storytelling with scientific content, AI-generated stories have the potential to become a powerful tool for fostering environmental awareness and responsibility in young children, contributing to the formation of environmentally responsible behaviors in future generations.

## Introduction

The rise of artificial intelligence (AI) technologies has opened new avenues in educational content creation, including the development of AI-generated stories (Hezam and Alkhateeb, 2024). These stories, designed to blend elements of art and science, offer unique opportunities to engage young children in learning by combining creativity with the presentation of complex concepts (Wu et al., 2020). For preschoolers, who are at a critical stage in their cognitive and emotional development, the use of AI-generated narratives can be a particularly effective method for introducing foundational ideas about environmental sustainability. By crafting stories that are enjoyable, engaging, and educational, AI-generated content can shape children’s pro-environmental attitudes and behaviors in ways that traditional methods may not (Guan et al., 2024).

The effectiveness of such interventions can be theoretically grounded in Bandura’s Social Learning Theory (Bandura and Walters, 1977), which posits that children acquire behaviors and attitudes through the observation and imitation of modeled actions, particularly when those models are perceived as attractive, competent, or similar to the observer. In this context, story characters—especially when consistently and positively represented—serve as symbolic models that demonstrate environmentally responsible behaviors.

Preschool-aged children are naturally curious and highly receptive to new information, making them ideal candidates for educational approaches that combine storytelling and environmental education. Stories have long been recognized as a powerful tool for conveying moral and social lessons, and recent advances in AI have enabled the creation of interactive and adaptive stories that respond to the needs and interests of individual children (Arn and Huang, 2024). In particular, AI-generated stories that blend art and science can simplify complex environmental concepts, such as climate change, biodiversity, and conservation, making them more accessible and engaging for young audiences. This interdisciplinary approach not only enhances children’s understanding of the natural world but also fosters emotional connections that are crucial for developing environmental sensitivity, a personal trait that influences their emotional and cognitive responses to nature.

Moreover, from a human-computer interaction perspective, children’s perception of the AI storyteller as a semi-social agent can influence their cognitive engagement and trust in the message (Breazeal, 2004; Reeves and Nass, 1996). Recent human-computer interaction research has highlighted that young users often attribute intentionality and credibility to interactive technologies, thereby increasing the persuasive power of the medium.

The enjoyment children derive from these stories is a key factor in their effectiveness (Chubb et al., 2022). When children enjoy a story, they are more likely to engage deeply with its content, internalize its messages, and reflect on the actions of its characters. In the context of AI-generated stories with pro-environmental themes, enjoyment can catalyze the development of pro-environmental attitudes and behaviors (Świątkowski et al., 2024). Research has shown that young children can develop environmental sensitivity through engaging and emotionally rich narratives (Choi and Nam, 2024). This environmental sensitivity, in turn, can mediate the relationship between the enjoyment of these stories and the likelihood of adopting pro-environmental behaviors, such as recycling or conserving resources (Dunne et al., 2024). In parallel, research on parasocial relationships has shown that young children often form emotionally meaningful and enduring bonds with media characters (Richards and Calvert, 2017), which enhances identification and increases the likelihood of modeling behavior. When the characters are familiar, prosocial, and environmentally conscious, these bonds can serve as important mechanisms for moral and ecological learning.

While existing studies have explored the potential of storytelling and environmental education to shape children’s behavior, the specific impact of AI-generated stories remains underexplored. This study aims to fill that gap by examining the influence of AI-generated stories that blend art and science on the pro-environmental attitudes and behaviors of preschoolers. The research focuses on three key hypotheses: (1) enjoyment of AI-generated stories will positively influence preschoolers’ pro-environmental behavior, (2) enjoyment of AI-generated stories will positively influence preschoolers’ environmental sensitivity, and (3) environmental sensitivity will mediate the relationship between story enjoyment and pro-environmental behavior. However, while prior research has examined the effects of storytelling and environmental education on young children, and other studies have explored the potential of AI-based tools in learning environments, no study to date has analyzed the specific role of AI-generated stories that blend art and science in shaping both environmental sensitivity and pro-environmental behavior in preschoolers. This gap in the literature underscores the need for empirical research examining how innovative narratives may influence environmental attitudes from an early age.

By investigating these relationships, this study seeks to contribute to the growing body of literature on the role of AI in early childhood education and its potential to shape environmentally responsible behaviors in the next generation.

In a world increasingly affected by environmental challenges, fostering pro-environmental attitudes from an early age is critical. AI-generated stories offer a novel and promising approach to environmental education for young children. Through carefully designed narratives that combine the artistic elements of storytelling with scientific content, these stories have the potential not only to educate but also to inspire preschoolers to become active participants in the protection and preservation of the environment.

## Literature review

### AI-generated stories blending art and science for preschoolers

AI-generated stories represent a promising educational tool for introducing preschoolers to environmental concepts. By leveraging storytelling—central in early education—these narratives can simplify complex ideas like sustainability into relatable messages (Blyler and Seligman, 2024; Pataranutaporn et al., 2021; Susanto et al., 2023). Evidence shows that AI-driven platforms can boost environmental awareness, with improvements of up to 30% among highly engaged children (Yumagulova et al., 2024), suggesting similar potential for preschool-targeted narratives. According to Bandura’s Social Learning Theory (Bandura and Walters, 1977), such stories can model pro-environmental behavior, using characters as symbolic guides for actions like recycling or protecting nature.

Preschoolers’ capacity for moral reasoning allows them to respond to stories depicting ecological values (Hu and Wu, 2022). When children witness characters benefiting from sustainable choices or facing consequences for harmful actions, they are more likely to internalize these lessons (Reed and Dodson, 2024). Parasocial relationships further enhance this process, as children tend to imitate admired characters (Bond and Calvert, 2014; Richards and Calvert, 2017; Hoffner and Buchanan, 2005). When integrated with Human-Computer Interaction principles, AI narratives gain credibility and engagement through interactive, agent-like behavior (Breazeal, 2004; Reeves and Nass, 1996).

In the Chinese context, where moral and environmental education are strongly emphasized, AI-generated stories can align with national educational goals by embedding culturally resonant themes and Confucian values like harmony and responsibility (Zhao et al., 2024; Guan et al., 2024). Customization allows these stories to reflect local realities—such as air pollution or water scarcity—enhancing relevance and promoting environmental stewardship at both local and global levels (Wu et al., 2020; Hezam and Alkhateeb, 2024).

Moreover, incorporating metacognitive elements into narratives—such as decision-making scenarios—can deepen reflection and foster a sense of agency in preschoolers (Hanisch and Eirdosh, 2023; Kaur and Saini, 2020; Rulli et al., 2024). These features encourage children to consider the consequences of their actions, reinforcing cognitive and emotional pathways to sustainable behavior.

Despite the rise of AI in education, no prior research has examined how AI-generated stories simultaneously influence environmental sensitivity and behavior in preschoolers. Addressing this gap, the present study explores how enjoyment of these narratives relates to both constructs, offering a novel interdisciplinary framework that integrates art, science, and environmental education.

### Enhancing environmental sensitivity through storytelling

Environmental sensitivity refers to the individual capacity to perceive and process stimuli from one’s surroundings, influencing emotional well-being, cognitive functioning, and behavior (Lionetti et al., 2024). While high sensitivity may pose challenges such as emotional reactivity or vulnerability to stress-related disorders like anxiety and depression (Assary et al., 2024), it can also be an asset in supportive environments (Islami et al., 2023). Emotional regulation plays a pivotal role in moderating these effects (Lionetti and Pluess, 2024).

Interactive storytelling—especially when enhanced by AI—can foster emotional engagement and empathy, two key mechanisms in cultivating environmental sensitivity. Digital narratives that explore issues like climate change or biodiversity loss help children form emotional connections with nature and its characters (Byman et al., 2022). These emotionally resonant experiences allow preschoolers to associate environmental care with positive affect, reinforcing long-term sensitivity and stewardship (MacKeen, 2022; Lannigan, 2022).

Technologies such as robotics further enrich this process. When embedded in storytelling, programmable robots can simulate real-world ecological scenarios—e.g., pollution or deforestation—creating hands-on, emotionally engaging experiences. Evidence suggests that such multisensory approaches enhance sensitivity, creativity, and emotional stability in young children (Arn and Huang, 2024; Choi and Nam, 2024).

Additionally, algorithmic elements integrated into AI-generated stories support problem-solving and reflective thinking. These narratives guide preschoolers through cause-effect environmental scenarios, enhancing both knowledge and sensitivity. Studies show that algorithmic technologies can increase high-level environmental understanding by up to 30% (Yumagulova et al., 2024).

In sum, AI-generated stories can powerfully enhance preschoolers’ environmental sensitivity by merging emotional engagement, interactive robotics, and cognitive reflection. These immersive narratives not only educate but also inspire care and responsibility toward the natural world.

### The mediating role of environmental sensitivity in AI-generated story enjoyment and pro-environmental behavior relationships

The enjoyment of AI-generated stories can positively influence preschoolers’ pro-environmental behavior, yet this effect is largely indirect and mediated by environmental sensitivity (Ding et al., 2024; Cheng and Wu, 2015). Children with heightened sensitivity to environmental issues tend to engage more deeply with eco-themed narratives, enhancing both emotional resonance and message internalization (Gong et al., 2020; Xiao et al., 2022). This engagement transforms story enjoyment into meaningful behavioral intentions.

Environmental sensitivity is a well-established predictor of pro-environmental behavior (Dunne et al., 2024). Sensitive children are more likely to perceive environmental challenges as personally relevant, leading to a stronger sense of responsibility and action orientation (Singh et al., 2022). AI-generated stories, when emotionally compelling and cognitively engaging, can activate this trait by linking enjoyment to reflective learning (Jain and Mitra, 2024).

Rather than undermining behavior, enjoyment can serve as a motivational gateway. When stories combine entertainment with environmental content, they foster positive affect, increase topic interest, and encourage sustainable actions—even in hedonic contexts (Liu and Chen, 2021). For children with higher environmental sensitivity, these narratives are more than just enjoyable—they become transformative learning experiences (Langenbach et al., 2020; Świątkowski et al., 2024).

This mediation process hinges on both emotional and cognitive pathways. Sensitive children not only empathize with nature-focused characters but also reflect on the long-term consequences of their actions (Guan et al., 2024). As such, environmental sensitivity deepens the link between story enjoyment and pro-environmental behavior by fostering awareness, responsibility, and agency (Liu and Green, 2024).

In summary, environmental sensitivity plays a central mediating role in translating enjoyment of AI-generated stories into pro-environmental behavior. By designing emotionally rich and pedagogically effective content, educators can activate this trait, encouraging preschoolers to adopt environmentally responsible habits from an early age.

**Figure 1:**
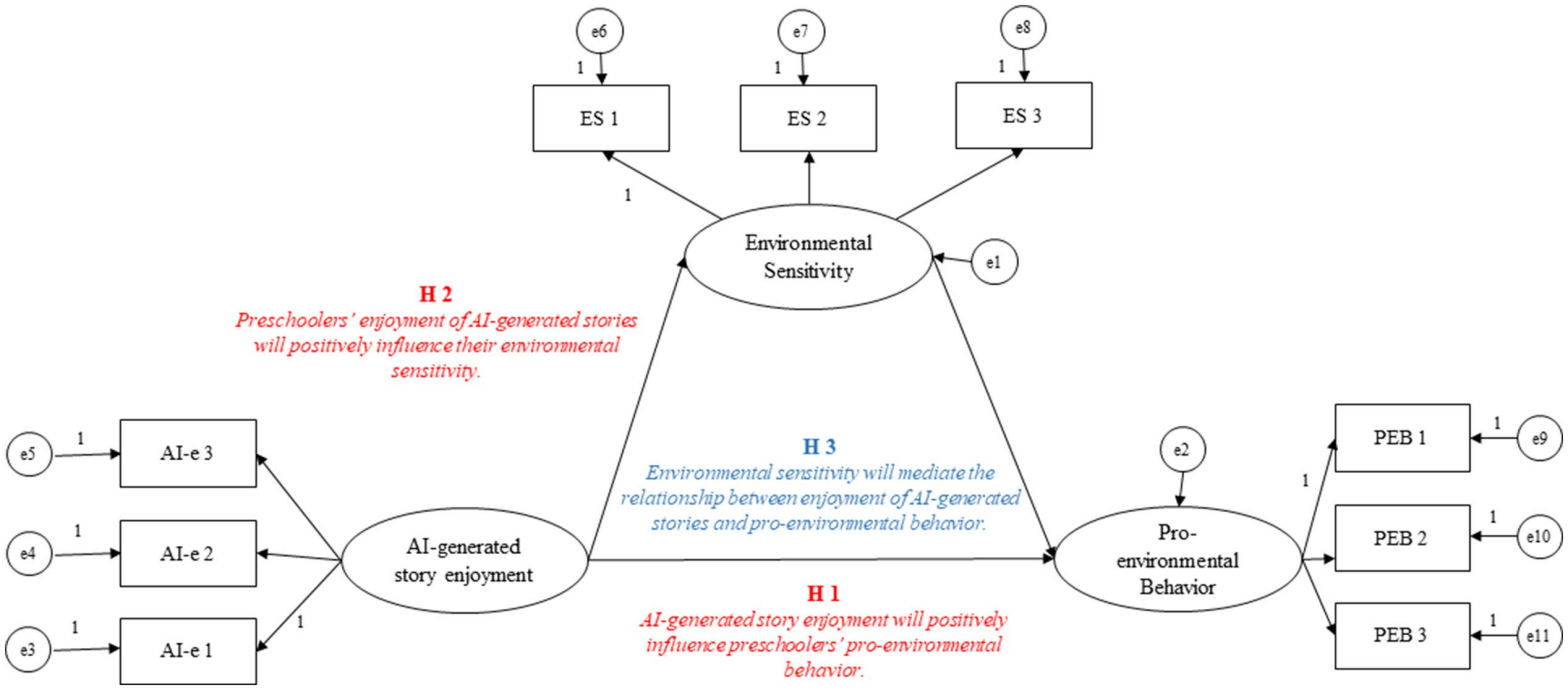
Tested the model of relationships between the study’s variables. AI-e: AI-generated story enjoyment; ES: Environmental Sensitivity; PEB: Pro-environmental Behavior. Direct relationships in red (H1 and H2) and indirect in blue (H3).

Figure 1 represents the tested model of relationships between the study’s variables.

In summary, this study investigates how preschoolers’ enjoyment of AI-generated stories blending art and science may influence their environmental attitudes and behavior.

The following three hypotheses are proposed:

H1: AI-generated story enjoyment will positively influence preschoolers’ pro-environmental behavior.

H2: Preschoolers’ enjoyment of AI-generated stories will positively influence their environmental sensitivity.

H3: Environmental sensitivity will mediate the relationship between enjoyment of AI-generated stories and pro-environmental behavior.

## Method

### Participants

The study included a total sample of 120 Chinese preschoolers from Upper Kindergarten (大班) in Jiangmen City, including its three urban districts (Pengjiang, Jianghai, Xinhui), Heshan, and the rural district Siyi. Children were at the final level of preschool education for children aged 5 to 6. Most of the participants, 66.7% (n = 80), were 6 years old, while 33.3% (n = 40) were 5 years old. This age distribution aligns with the typical age range for children in Upper Kindergarten, with a greater emphasis on those aged six who are nearing the transition to primary school.

Regarding gender, the sample had more females than males. Specifically, 61.7% (n = 74) of the participants were female, and 38.3% (n = 46) were male, indicating a slight gender imbalance with a predominance of girls.

The preschoolers were also distributed across six different classes. The largest group came from class b, comprising 21.7% (n = 26) of the sample, while the smallest group came from class a, with 11.7% (n = 14). The remaining children were fairly evenly distributed across the other classes, with 20.0% (n = 24) in class c, 13.3% (n = 16) in class d, and 16.7% (n = 20) in both class e and class f. This class distribution ensures a broad representation of children from different classroom environments within the Upper Kindergarten program.

### Procedure

The study followed a carefully structured procedure to ensure ethical standards and maximize the reliability of data collection while keeping the children engaged and avoiding fatigue. Ethical approval for the study was obtained from the Ethical Committee of Jiangmen Preschool Education College (Approval number 8980-EDU-57, date of approval 4^th^ April 2024), and informed consent was acquired from the parents or legal guardians of all participating children. In the informed consent, the parents or legal guardians received a brief overview of the study, an information bulletin, and a consent form. The study used a panel design with multiple assessments spread over three time points, separated by 1 week, to maintain children’s interest and prevent boredom. Information regarding each child’s age and gender was provided by their classroom teachers, who compiled this information from school records. This approach allowed for accurate demographic categorization while maintaining confidentiality and minimizing burden on families. No data were collected regarding family income or parental education, in accordance with institutional ethical guidelines that prioritized the protection of children’s privacy and avoided intrusive questions in the context of early childhood education. These decisions were aligned with the study’s educational setting and the restrictions outlined in the approved research protocol.

At the first point (T1, second week of May 2024), children were introduced to the AI-generated stories. After listening to the stories, their level of enjoyment was assessed. At the second time point (T2, third week of May 2024), environmental sensitivity was measured through a game designed to gauge children’s awareness and sensitivity to environmental issues. At the third and final time point (T3, fourth week of May 2024), children’s pro-environmental behavior was assessed through a task aimed at evaluating their actions and attitudes related to environmental conservation.

Each child’s class teacher administered the tasks to ensure that the assessments were carried out effectively. Prior to the first assessment, all teachers underwent comprehensive training to familiarize themselves with the process and ensure consistent delivery across the classrooms. During the sessions, teachers read the questions aloud to the students, ensuring that all children, regardless of reading ability, could understand and respond to the questions. This approach was particularly important for younger children who may have had difficulty reading on their own.

To ensure the standardization and fairness of the testing environment, only the children whose parents or legal guardians had provided informed consent remained in the classroom during each session. Children who did not have parental consent were temporarily withdrawn from the room during the assessment, thus preventing any unintended peer influence or perceived unequal treatment.

Strict protocols were followed during the questionnaire sessions to ensure individual responses. Students were instructed to complete the assessments independently, without interacting with their classmates, to prevent any potential influence or sharing of answers. These measures were implemented to maintain the integrity of the data and ensure that the responses truly reflected each child’s individual experience and understanding.

### Instruments

*AI-generated story blending arts and sciences enjoyment* (Time 1). The short story was generated by ChatGPT in March 2024 using the prompt: “Please write me a short story for 5-year-old preschoolers blending art and science with a focus on environmental protection.” Initially, the story was less than 300 words. To enhance it, I asked ChatGPT to “retell the story to include avoiding water pollution, promoting animal protection, and addressing air pollution.” This revision kept the same basic plot but included more details about these three environmental concerns, resulting in a longer version (467 words). Please see the Appendix for the AI-generated story.

To measure the children’s enjoyment of the AI-generated story blending art and science with an environmental protection theme, a 5-point visual analogue scale featuring expressive faces was employed. This method has proven effective in child-centered research, particularly in measuring subjective emotional states and enjoyment in children (Mispa et al., 2016). The scale ranged from a very frowning face (representing “really did not like it”) to a very smiling face (representing “loved it”). Each face corresponded to one of the following five points:

- Very frowning face—really did not like it
- Slightly frowning face—did not like it much
- Neutral face—it was okay
- Slightly smiling face—liked it
- Very smiling face—loved it

After listening to the story, each child was individually shown the visual scale and asked the question: “Which face shows how you felt about the story?” phrased in simple, age-appropriate language. To ensure comprehension, a practice round using a different topic was conducted prior to the actual evaluation. Large, clearly printed visual aids of the faces were displayed to support identification, and responses were collected immediately after the story, while the experience was still recent. To minimize external influences, responses were obtained one-on-one, preventing peer pressure or group conformity. Additionally, consistent administration procedures were used for all participants. The use of visual, non-verbal scales has been shown to be both reliable and developmentally suitable for assessing preschoolers’ preferences and affective responses (Roberto et al., 2010; Salvador-Herranz et al., 2013; Tatla, 2014).

This method is widely endorsed in child-computer interaction and educational studies for its ability to accurately reflect young children’s subjective experiences, including enjoyment and motivation, in a non-verbal yet quantifiable manner (Mispa et al., 2016).

*Environmental sensitivity* (Time 2) was assessed using a simplified version of Game 1b from the Games Testing Tool for Preschoolers, originally developed by Giusti et al. (2014) and later adapted by other researchers (Omidvar et al., 2019a, 2019b). This tool was further modified to suit the developmental and geographic needs of Canadian preschoolers in the study by MacKeen and Wright (2020). The psychometric properties of this instrument were examined by MacKeen et al. (2022). Specifically, the Game 1b component demonstrated an individual content validity index (I-CVI) of 0.833, which exceeds the commonly recommended cutoff of 0.80. However, it should be noted that some global scale indices were below optimal thresholds.

For the current study, this adapted version was applied to measure environmental sensitivity in 5- to 6-year-old children. The task involved a simple, engaging game in which children were shown a series of pictures and asked to place either a “happy” or “sad” face on top of each image, depending on how they felt about it. The pictures included scenarios related to environmental issues, such as dirty water, smoky air, and cleaning up. For instance, an appropriate response would be placing a happy face on an image depicting cleaning up, while an incorrect response might be placing a sad face on the same image. Correct responses were taken as indicators of environmental sensitivity, while incorrect responses reflected a lack of sensitivity to environmental issues.

To administer the task, the researcher explained the activity to the child, using simple language and demonstrating the process. For example, they might say, “We are going to play a game of happy and sad faces, and I would like you to put a happy smile or sad face on each photo you see here.” The child was then given the option to choose between a happy or sad face cutout and asked to place their choice on each picture. A total of 15 face cutouts were printed, laminated, and enlarged to make the activity more interactive and engaging for the children. The researcher held up the five faces for each picture, allowing the child to decide which face to place over each image without explaining the meaning of the pictures or influencing their decisions. For example, while pointing to a picture of dirty water, the researcher would ask, “Which smiley would you like to place there?” The child then made their choice, and the researcher recorded their response on a scoresheet. An adapted 5-point scale was used to gauge the child’s responses. This scale was similar to the one used at the first time. Correct responses, such as choosing a happy face for cleaning up, were considered as evidence of environmental sensitivity. Incorrect responses, such as a sad face for the same scenario, indicated lower environmental sensitivity. This method provided a simple yet effective way to assess each child’s awareness and attitudes toward environmental issues without requiring verbal explanation or influencing their choices.

*Pro-environmental behavior* was assessed using a modified version of the Scale of Preschool Children’s Pro-Environmental Attitudes (Li et al., 2024), which originally measured children’s preferences for pro-environmental behaviors through pictorial representations. The full scale contains 11 items, but for this study, three specific dimensions were used: air pollution, water pollution, and animal protection. Each dimension was represented by a series of line drawings depicting either environmentally unfriendly or pro-environmental behaviors.

For each item, the children were presented with simple line drawings, and the research assistant read descriptions of the behaviors depicted in the images. For example, for water conservation, the assistant would describe one image as “The man washed his hands, but he did not turn off the water” and another as “The man washed his hands and turned off the water.” These drawings, along with their descriptions, allowed children to distinguish between pro-environmental and environmentally unfriendly behaviors easily.

The children were then asked to indicate their preferences for each drawing by selecting one of five faces on a scale. The response options were the same used in the first and second times.

For each of the three dimensions (air pollution, water pollution, and animal protection), one drawing depicted an environmentally unfriendly behavior, which was scored in reverse. In contrast, the other two drawings depicted pro-environmental behaviors. For instance, in the dimension of animal protection, one image showed people hunting and killing animals, which was scored in reverse. The research assistant guided the children by asking, “Do you like the drawing a little bit or a lot?” to prompt them to select their preferred face on the scale. This method ensured that children could express their attitudes toward the depicted behaviors in a way that was easy to understand and engage with.

### Debriefing

Debriefing was conducted only after the final assessment session (T3), in order to avoid influencing children’s responses during earlier phases of the study. After completing the game-based assessments, the researcher debriefed each child by explaining the concept of pollution in simple, age-appropriate terms. The debriefing began with an introduction to the topic, “Today, we talked about different types of pollution. Now, I’ll go over these ideas with you. If you have any questions, feel free to ask.” The researcher then discussed water pollution, explaining that it occurs when waste and chemicals enter bodies of water like oceans or rivers, making the water unsafe for fish and other animals. Next, air pollution was introduced by describing how harmful gases and chemicals in the air can create smog or a smoky appearance, which can lead to health problems for both animals and humans, such as difficulty breathing. Throughout the debriefing, the researcher encouraged the child to ask questions to ensure they understood the information. At the end of the session, the researcher made sure to check again if the child had any further questions or thoughts.

### Data analyses

First, descriptive statistics, including means and standard deviations, were calculated for the key variables, and correlational analyses were performed using Pearson’s correlation coefficients to examine the relationships between the variables using the SPSS 29.0 version. To explore the direct and indirect effects of AI-generated story enjoyment on pro-environmental behavior, structural equation modeling (SEM) was used with AMOS (Version 29.0). SEM allowed for the simultaneous testing of multiple relationships, including the potential mediating role of environmental sensitivity. The model fit was assessed using several indicators, such as the chi-square to degrees of freedom ratio (CMIN/DF). Given that chi-square is sensible to the sample size, other fit indices are recommended. Hence, the study relied on the root mean square error of approximation (RMSEA), comparative fit index (CFI), and goodness-of-fit index (GFI). These indices were used to evaluate how well the proposed model fits the data. Finally, regression analyses were conducted to estimate the direct, indirect, and total effects of AI-generated story enjoyment on environmental sensitivity and pro-environmental behavior. The results included standardized regression coefficients (beta values), standard errors, and significance levels. The indirect effects were examined to assess the mediating role of environmental sensitivity in the relationship between story enjoyment and pro-environmental behavior. Throughout the analysis process, steps were taken to ensure consistency and independence in responses, maintaining the reliability and validity of the results.

## Results

### Descriptive and correlational analyses

The means, standard deviations, and sample size for the variables included in the analysis are summarized. The variable AI-generated story enjoyment showed a relatively high level of enjoyment of AI-generated stories among the participants. Environmental Sensitivity had a slightly lower mean score, indicating moderate sensitivity to environmental issues among preschoolers. Pro-environmental Behavior exhibited a moderate mean score, suggesting a tendency toward environmentally friendly behavior among the children.

Correlational analyses revealed that the three key variables—AI-generated story enjoyment, environmental sensitivity, and pro-environmental behavior—were all significantly and positively associated with one another. These results support the expected directional relationships among enjoyment, sensitivity, and environmentally responsible actions in preschoolers (Table 1).

**Table 1:** Descriptive statistics and Pearson’s correlation matrix (*N* = 120).

Variable	M	SD	1	2	3
1. AI-generated stories Enjoyment	3.92	0.76	—		
2. Environmental Sensitivity	3.73	0.74	0.40**	—	
3. Pro-environmental Behavior	3.23	0.40	0.28**	0.48**	—

*p* < 0.01.

#### Hypotheses testing

The first hypothesis (H1) proposed that AI-generated story enjoyment would positively influence preschoolers’ pro-environmental behavior. Structural equation modeling (SEM) was employed to test this hypothesis.

The model was evaluated for overall fit using various indices. The chi-square value was significant, χ^2^ (24) = 41.96, *p* = 0.013, suggesting some misfit between the model and the data. However, the chi-square to degrees of freedom ratio (CMIN/DF = 1.749) was below the recommended threshold of 3, indicating an acceptable fit. The model showed acceptable fit: GFI = 0.936, AGFI = 0.881, RMR = 0.041, CFI = 0.963, and RMSEA = 0.079 (90% CI [0.036, 0.118]).

The regression results showed that AI-generated story enjoyment had a significant positive effect on pro-environmental behavior (β = 0.517, *p* < 0.001). This suggests that preschoolers who enjoy AI-generated stories are more likely to engage in pro-environmental actions. Additionally, AI-generated story enjoyment significantly influenced environmental sensitivity (β = 0.468, p < 0.001), supporting the idea that higher enjoyment increases children’s awareness and sensitivity toward environmental issues.

The path from environmental sensitivity to pro-environmental behavior was marginally significant (β = 0.211, *p* = 0.054). This result indicates that while environmental sensitivity plays a role in shaping pro-environmental behavior, its influence may be weaker than direct enjoyment of the stories. Overall, the results support H1, demonstrating that the enjoyment of AI-generated stories blending art and science positively influences both environmental sensitivity and pro-environmental behavior in preschoolers, as Table 2 shows.

**Table 2:** Regression analysis results.

Predictor	B	SE	β	CR	p
AI-generated story enjoyment → Environmental Sensitivity	0.317	0.081	0.468	3.923	< 0.001
Environmental Sensitivity → Pro-environmental Behavior	0.242	0.126	0.211	1.927	0.054
AI-generated story enjoyment → Pro-environmental Behavior	0.403	0.090	0.517	4.459	< 0.001

B = unstandardized regression coefficient, SE = standard error, β = standardized regression coefficient, CR = critical ratio (*z*-value), *p* = significance level.

Hypothesis 2 (H2) proposed that preschoolers’ enjoyment of AI-generated stories would positively influence their environmental sensitivity. The results from the structural equation modeling (SEM) support this hypothesis. The regression analysis revealed that AI-generated story enjoyment had a significant positive effect on environmental sensitivity (B = 0.317, SE = 0.081, β = 0.468, CR = 3.923, *p* < 0.001). This indicates that as preschoolers’ enjoyment of AI-generated stories increases, their sensitivity to environmental issues also increases. These findings suggest that the more children enjoy the stories blending art and science, the more aware and sensitive they become to environmental concerns. This supports the idea that engagement with AI-generated educational content can positively influence children’s attitudes toward the environment, fostering greater environmental awareness at an early age. The significant positive relationship between enjoyment and environmental sensitivity provides strong support for Hypothesis 2, as Table 2 shows.

Hypothesis 3 (H3) proposed that environmental sensitivity would mediate the relationship between enjoyment of AI-generated stories and pro-environmental behavior. To test this, the total, direct, and indirect effects of AI-generated story enjoyment on pro-environmental behavior were examined through structural equation modeling (SEM).

The total effect of AI-generated story enjoyment on pro-environmental behavior was significant (B = 0.480, β = 0.616). This total effect reflects both the direct and indirect pathways through which AI story enjoyment influences pro-environmental behavior. The significant total effect suggests that enjoyment of AI-generated stories has a strong influence on children’s pro-environmental actions.

The direct effect of AI-generated story enjoyment on pro-environmental behavior was also significant (B = 0.403, β = 0.517, p < 0.001). This indicates that a substantial portion of the effect of story enjoyment on pro-environmental behavior occurs without mediation, meaning that children who enjoy the stories are directly more likely to engage in pro-environmental behaviors.

Additionally, the direct effect of environmental sensitivity on pro-environmental behavior was marginally significant (B = 0.242, β = 0.211, *p* = 0.054). This suggests that environmental sensitivity plays a role in shaping pro-environmental behaviors, although the effect is weaker compared to the direct effect of AI story enjoyment.

The indirect effect of AI-generated story enjoyment on pro-environmental behavior through environmental sensitivity was also examined. The results showed a significant indirect effect (B = 0.077, β = 0.099), suggesting that environmental sensitivity partially mediates the relationship between AI-generated story enjoyment and pro-environmental behavior. This implies that while enjoyment of the stories directly influences behavior, it also fosters environmental sensitivity, which, in turn, promotes pro-environmental actions.

The results support Hypothesis 3, showing that environmental sensitivity partially mediates the relationship between AI-generated story enjoyment and pro-environmental behavior. However, the direct effect of story enjoyment remains stronger, indicating that while environmental sensitivity is an important mediator, the enjoyment of the stories has a more pronounced influence on children’s pro-environmental behaviors (Table 3).

**Table 3:** Total, direct, and indirect effects.

Effect Type	Path	B	β	p
Total Effect	AI-generated story enjoyment → Pro-environmental Behavior	0.480	0.616	< 0.001
Direct Effect	AI-generated story enjoyment → Pro-environmental Behavior	0.403	0.517	< 0.001
Direct Effect	Environmental Sensitivity → Pro-environmental Behavior	0.242	0.211	0.054
Indirect Effect	AI-generated story enjoyment → Environmental Sensitivity → Pro-environmental Behavior	0.077	0.099	0.054

B = unstandardized coefficient, β = standardized coefficient, *p* = significance level.

**Figure 2:**
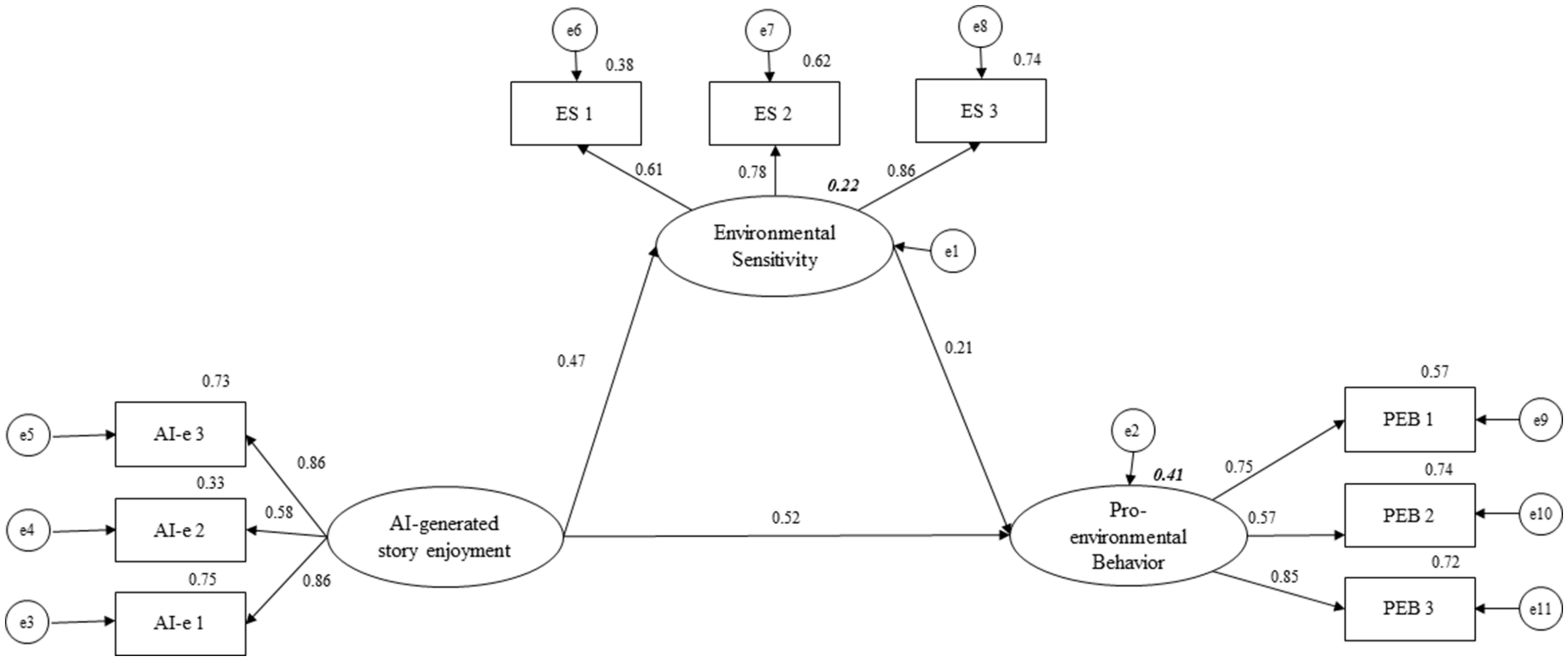
SEM model with the standardized paths. AI-e: AI-generated story enjoyment; ES: Environmental Sensitivity; PEB: Pro-environmental Behavior. Values above the arrows are the standardized paths or factor loadings. Values in bold, italics above the variables are the r-squared.

Figure 2 displays the SEM model with the standardized paths.

## Discussion

The findings of this study contribute to the growing body of literature on the intersection between technology, education, and environmental psychology, particularly in the context of young children’s learning experiences.

### AI-generated story enjoyment positively influences preschoolers’ pro-environmental behavior (H1)

In line with the first hypothesis, the present results demonstrate that AI-generated story enjoyment significantly enhances pro-environmental behavior among preschoolers. This finding aligns with previous research indicating that enjoyment is a crucial factor in fostering positive learning outcomes in children, particularly when educational content is presented through engaging narratives and multimedia formats (Mares and Woodard, 2005). However, the current study extends this understanding by applying it specifically to AI-generated content blending art and science, which has not been widely explored in the existing literature.

The positive relationship between enjoyment and pro-environmental behavior suggests that emotional engagement with educational content, particularly through storytelling, can be a powerful tool for shaping children’s attitudes and behaviors toward environmental issues. This is consistent with findings from research on environmental education, which highlights the importance of affective factors, such as emotional connections to nature, in fostering pro-environmental attitudes (Chawla, 1998). In this context, AI-generated stories may provide a novel and interactive way to evoke emotional responses that motivate pro-environmental behavior in young children.

This discrepancy could be due to developmental differences in cognitive and emotional processing between preschoolers and older populations. Younger children may respond more strongly to immediate emotional engagement than to more abstract constructs such as environmental sensitivity. This finding aligns with Choi and Nam (2024), who demonstrated that emotionally rich storytelling experiences, especially when enhanced through technology such as educational robotics, significantly foster environmental sensitivity and creativity in 5-year-old children. Furthermore, recent evidence by Albrecht et al. (2024) indicates that enjoyment-driven contexts, such as leisure activities, do not necessarily inhibit pro-environmental behavior—particularly when children already have positive environmental habits or exposure. Langenbach et al. (2020) also suggest that enjoyment enhances engagement with environmental content, provided that sufficient cognitive resources are available. Taken together, these insights support the notion that in early childhood, emotionally engaging experiences—such as AI-generated stories—may function as a more immediate and effective driver of pro-environmental behavior than reflective sensitivity alone.

### Preschoolers’ enjoyment of AI-generated stories positively influences their environmental sensitivity (H2)

The results supporting Hypothesis 2 offer important insights into the role of emotional engagement in environmental education, particularly through the use of AI-generated stories blending art and science. The finding that preschoolers’ enjoyment of these stories positively influences their environmental sensitivity underscores the significance of affective experiences in shaping young children’s attitudes toward environmental issues. This finding aligns with the broader literature on environmental education, which emphasizes the importance of emotional connection and engagement as key factors in fostering environmental sensitivity (MacKeen et al., 2022). However, this study extends that body of knowledge by exploring the unique potential of AI-generated content in this domain.

The positive relationship between enjoyment and environmental sensitivity suggests that AI-generated stories may function as an effective tool for raising environmental awareness in young children by leveraging their natural inclination for play and imagination. These stories, which combine artistic and scientific elements, appear to stimulate both cognitive and emotional engagement, making environmental topics more accessible and relevant to preschoolers. This supports previous research suggesting that interactive and enjoyable learning experiences are particularly effective at promoting environmental awareness in early childhood (Albrecht et al., 2024).

What sets this study apart from previous work is the specific focus on AI-generated stories as a medium for environmental education. While traditional forms of storytelling and multimedia have been well-documented as powerful educational tools, the current findings suggest that AI-generated content can similarly evoke emotional responses that enhance children’s sensitivity to environmental issues (Choi and Nam, 2024). This aligns with emerging research on the use of AI in educational settings, which has demonstrated that AI-generated content can be both engaging and effective in promoting positive learning outcomes (Kang and Jang, 2024). Moreover, recent studies have emphasized that individuals with higher environmental sensitivity tend to develop stronger emotional connections to nature, making them particularly responsive to emotionally resonant educational stimuli (Dunne et al., 2024; Pluess et al., 2023). Our findings are consistent with this view, highlighting that affective engagement—through enjoyment—may be especially powerful for fostering early sensitivity.

At the same time, the significant positive relationship between enjoyment and environmental sensitivity adds nuance to our understanding of how emotional engagement with educational content can influence young children’s development. Previous research has often focused on the role of cognitive factors, such as knowledge acquisition, in shaping environmental sensitivity (Barbas et al., 2009). However, the current study suggests that emotional factors, particularly enjoyment, may play an equally if not more critical role, especially for younger children who may not yet have developed the cognitive capacity to understand complex environmental issues fully.

To our knowledge, no prior research has examined the role of enjoyment in AI-generated stories as a predictor of environmental sensitivity in preschoolers. This combination of affective engagement and technological storytelling represents a novel educational strategy for early sensitivity formation. While the current study focused on preschoolers, it is possible that similar results could be observed in older children or even adults, given the growing body of literature suggesting that enjoyment and emotional engagement are key drivers of environmental attitudes across the lifespan (Kong and Jia, 2023). Further research is needed to explore the extent to which AI-generated content can foster environmental sensitivity in different populations and to investigate the mechanisms through which this occurs.

### Environmental sensitivity mediates the relationship between enjoyment of AI-generated stories and pro-environmental behavior (H3)

The findings related to Hypothesis 3 provide important insights into the complex interplay between enjoyment, environmental sensitivity, and pro-environmental behavior in preschoolers. The results confirm that environmental sensitivity mediates the relationship between AI-generated story enjoyment and pro-environmental actions. However, the direct effect of enjoyment remains more dominant, suggesting that emotionally engaging stories are a more immediate driver of behavior in early childhood. This supports findings by Pluess et al. (2023) and (Choi and Nam, 2024), who emphasize that emotional connection is a crucial precursor to environmental awareness and action in young children. Moreover, our results are consistent with Dunne et al. (2024), who demonstrated that sensory processing sensitivity, linked with enjoyment and nature connectedness, predicts future-oriented environmental behavior.

While enjoyment has a more immediate effect, the partial mediation by environmental sensitivity indicates that fostering deeper awareness and emotional connection still plays a key role in sustaining pro-environmental intentions. As Singh et al. (2022) observed, environmental sensitivity contributes to environmental behavior through a heightened sense of moral responsibility. In preschoolers, this pathway may begin with story-driven emotional engagement that progressively activates early sensitivity.

At the developmental level, our results suggest that enjoyment may be especially potent in early childhood due to limited abstract reasoning. Compared with studies in older children and adolescents—where sensitivity often mediates more robustly (Wu, 2018)—this finding supports stage-based educational approaches that begin with emotionally rich content and gradually introduce more complex environmental concepts.

Enjoyment alone does not inhibit pro-environmental behavior. On the contrary, Albrecht et al. (2024) found that enjoyment-linked activities, such as tourism, can still foster sustainable behavior when supported by personal effort and habit. Similarly, Langenbach et al. (2020) showed that enjoyment enhances attention and motivation toward environmental content, provided that cognitive resources are present. For preschoolers, the design of AI-generated stories must therefore balance emotional engagement with age-appropriate cognitive input.

Beyond psychological mechanisms, ethical considerations are increasingly important. As other authors (Rismayati et al., 2019; Wu, 2018) emphasized, AI in education must support sustainability not only in message but also in practice—ensuring inclusivity, transparency, and cultural relevance. These considerations are crucial when deploying AI to shape the worldview of young learners.

In summary, the present findings clarify that while environmental sensitivity partially mediates the relationship between AI-generated story enjoyment and pro-environmental behavior, enjoyment remains the primary driver at the preschool stage. These results reinforce the role of affective engagement as a catalyst for early environmental behavior, and they position AI-generated stories as a promising tool to both activate sensitivity and promote action. To realize this potential, future designs of AI educational content should prioritize emotional resonance, developmental fit, and ethical transparency, thus maximizing both educational impact and long-term environmental stewardship.

### Practical applications and ethical consideration of AI use

This study highlights how AI-generated educational content can foster environmental responsibility in early childhood by combining enjoyment with learning. When designed thoughtfully, these stories offer personalized, engaging experiences that promote sustainable habits in young children (Jain and Mitra, 2024). However, emotional engagement is critical—especially at preschool age—since narratives that lack affective resonance may fail to inspire behavioral change (Guan and Geng, 2024). Thus, story design should prioritize emotional relevance alongside interactivity and informativeness.

Adaptive technologies, such as collaborative filtering, can tailor AI-generated stories to developmental needs (Zhang, 2025), enhancing both engagement and educational impact. Research confirms that enjoyment, when balanced with cognitive clarity, deepens children’s connection to environmental themes (Langenbach et al., 2020; Albrecht et al., 2024). Stories with emotionally relatable characters and simple, actionable messages are particularly effective at this stage of development.

AI-generated stories may also replicate benefits traditionally offered by real-life experiences, such as outdoor learning. As shown in Chilean early education settings, emotionally immersive environments foster environmental awareness (Christie et al., 2025). Beyond education, environmental sensitivity is gaining relevance in fields like social work and urban design, where it informs training programs and interventions (Besthorn, 2012; Avinç and Doğan, 2025; Jeon et al., 2010).

Nevertheless, ethical considerations are crucial. AI-generated content must deliver scientifically accurate information and avoid oversimplification or misinformation, which can distort children’s understanding (Holmes et al., 2019). Transparency, human oversight, and algorithmic accountability are essential to preserve educational integrity.

Equally important is the promotion of diversity and inclusion. AI systems must avoid reinforcing harmful stereotypes and instead reflect pluralistic values (Hendricks and Vestergaard, 2019). Content should be culturally relevant and free from bias to ensure equitable learning experiences.

In sum, AI-generated stories offer transformative potential in environmental education, blending emotional engagement with cognitive development. To realize this promise, developers and educators must embrace ethical principles—accuracy, transparency, inclusivity—and design content that not only educates but also inspires a sustainable mindset from early childhood (Ma, 2023).

## Limitations and conclusion

This study offers valuable insights into how AI-generated story enjoyment relates to environmental sensitivity and pro-environmental behavior in preschoolers; however, some limitations must be acknowledged. First, the geographically limited sample (120 children from Jiangmen City, southern China) restricts generalizability across cultural and socioeconomic contexts. Future studies should include more diverse populations to assess the robustness of these findings (Ahmad et al., 2022; Danesi, 2024). Second, the age range (5 to 6 years) reflects only a specific developmental stage; broader age groups are needed to understand how developmental maturity influences environmental responsiveness (Assary et al., 2024). Third, the gender imbalance (61.7% girls) may have influenced results, especially given prior findings suggesting higher environmental sensitivity in girls (Stinson, 1985); future samples should aim for gender balance.

Additional limitations relate to methodological and contextual factors. Although the visual and interactive measures were appropriate, they may not fully capture the complexity of environmental sensitivity or behavioral intentions (Langenbach et al., 2020; MacKeen et al., 2022). The short, three-week duration limits conclusions on long-term behavioral change, underscoring the need for longitudinal designs. Moreover, delayed debriefing may have reduced the immediate educational impact of the tasks. Finally, the study did not account for external influences such as family attitudes, curricula, or community programs (Jia and Yu, 2021; Zheng and Sun, 2023), which may shape environmental development. Future research should adopt more ecological frameworks that integrate these broader variables.

In sum, AI-generated stories show promise for fostering environmental sensitivity and pro-environmental behavior in early childhood education. Yet, their implementation must be guided by ethical principles that ensure inclusivity, transparency, and contextual relevance. When thoughtfully integrated, AI can contribute not only to early environmental awareness but also to a more equitable and sustainable future (Husin et al., 2025).

## Data Availability

The raw data supporting the conclusions of this article will be made available by the authors, without undue reservation.

## Appendix

### AI-generated short story blending arts and sciences

Once upon a time, in a bustling town called 彩虹镇 (Cǎihóng Zhèn—Rainbow Town), lived a curious little girl named 莉莉 (Lìlì—Lily). She loved everything colorful, especially rainbows. She loved painting rainbows, making rainbow paper cuttings, and even rainbow-colored dumplings!

One sunny afternoon, while painting in her courtyard, Lily noticed something unusual. The sky was gray, the flowers drooped, and even the crickets seemed quiet. The nearby river, once teeming with life, now flowed sluggishly, its surface marred by floating debris. But what saddened Lily the most was the absence of the birds and butterflies that used to fill the town with their songs and colors. Lily’s heart ached. Where had all the colors and life gone?

She asked her 奶奶 (Nǎinai—grandma), who explained that pollution from factories, cars, and people not taking care of the environment was making everything sad. The air was dirty, the water was polluted, and even the animals were struggling to find food and clean homes.

Lily did not like the sound of that. She wanted to bring the colors and life back! So, she decided to use her love for art and science to help. With her grandma’s help, Lily started collecting recyclable materials like bottles, cans, and old newspapers. She cleaned them, painted them with vibrant colors, and turned them into beautiful art pieces. She made rainbow lanterns from bottles, colorful planters from cans, and even a giant rainbow dragon from old newspapers.

But Lily also wanted to help the animals. She built bird feeders and insect hotels from recycled materials, filling them with seeds and creating cozy spaces for the creatures to rest. She even painted signs reminding people not to litter and to be kind to animals.

Lily invited all her friends to join her. They transformed their town into a colorful wonderland full of recycled art and eco-friendly initiatives. Soon, the gray sky started turning blue, the flowers bloomed again, and the crickets chirped happily. The river sparkled once more, and the animals returned to their happy homes, their songs and colors filling the town with joy.

The townspeople were amazed at what Lily and her friends had achieved. They learned that even small actions, like recycling, reducing pollution, and caring for animals, could make a big difference.

Lily’s story spread far and wide, inspiring others to use their creativity and knowledge to protect the environment and all its creatures. 彩虹镇 (Cǎihóng Zhèn) became known as the town where art and science met to create a brighter, greener future for everyone. And Lily, the little girl who loved rainbows, became a symbol of hope and inspiration, reminding everyone that even the smallest hands can make the world a more beautiful and healthy place for all living things.
